# Supplementary material for: A spoonful of L‐fucose—an efficient therapy for GFUS‐CDG, a new glycosylation disorder
Source: EMBO Mol Med. 2021 Sep 1;13(9):e14332. doi: 10.15252/emmm.202114332 (PMC8422078; doi:10.15252/emmm.202114332)
Supplement: Supplementary file 1 — Expanded View Figures PDF [file EMMM-13-e14332-s006.pdf]

# Expanded View Figures

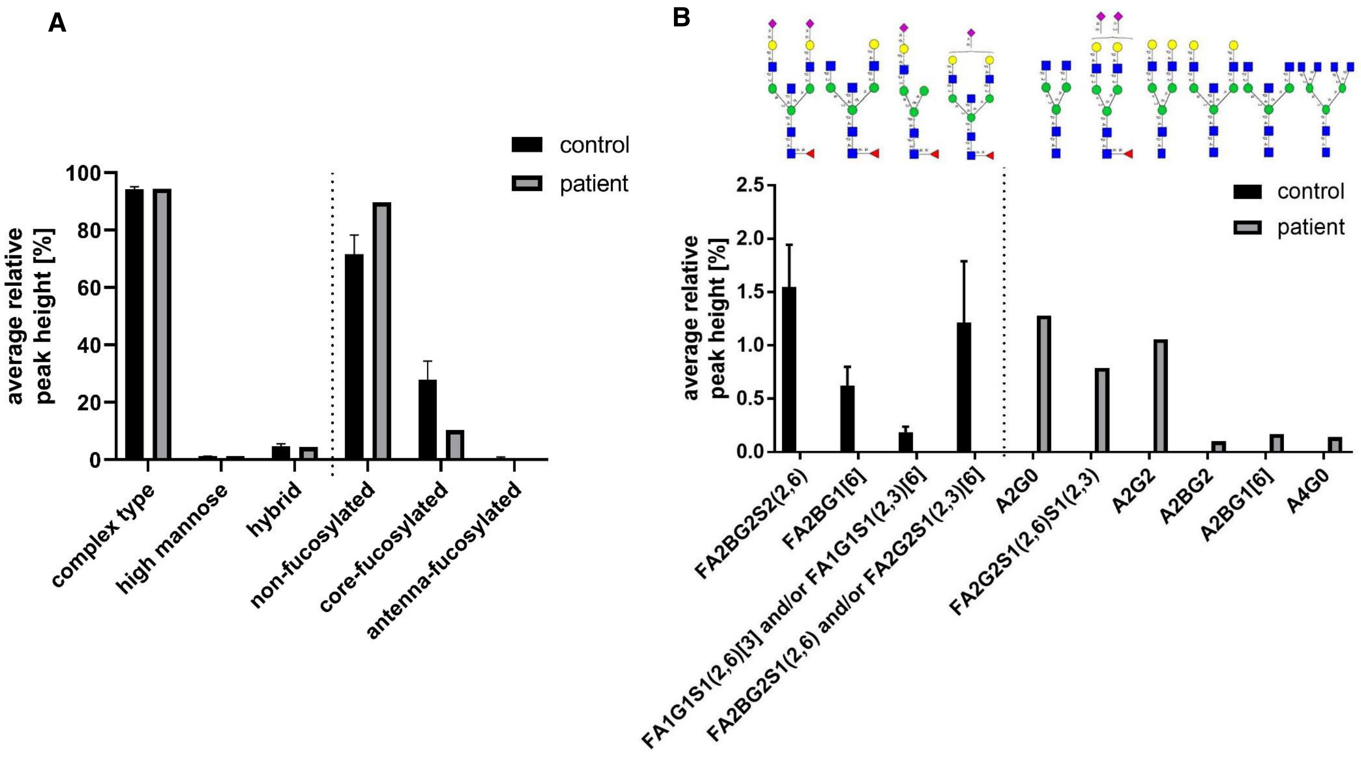


**Figure EV1. Analysis of N-glycans from whole serum glycoproteins.**

To investigate the general hypofucosylation in the patient, released serum N-glycans were measured by xCGE-LIF. (Data were obtained from serum; $n = 10$ for controls; experiment was independently repeated two times).

A Change of relative N-glycan composition. While the patient's distribution of hybrid-, high-mannose and complex type N-glycan was not altered, a marked reduction of core- and antenna-fucosylated structures was detected in comparison to the controls.

B Unique glycans. Several unique glycans were identified either in the control pool as well as in the patient-derived serum. Whether one of them can serve as biomarker for GFUS-CDG needs to be determined.

Data information: N-glycan structures were produced using Glycan Builder 2 (Tsuchiya *et al*, 2017) following the Symbol Nomenclature for Glycans (SNFG) guidelines (Neelamegham *et al*, 2019). Naming of N-glycan structures was adapted from the Oxford nomenclature (Doherty *et al*, 2018).

 © 2021 The Authors

**Figure EV2. Aleuria aurantia lectin staining of blood smears and FACS analysis from the affected individual, the mother and an additional control.**

A Merge of lectin (green) and DAPI (blue) staining of a control.
B Merge of lectin (green) and DAPI (blue) staining of the heterozygous mother.
C Merge of lectin (green) and DAPI (blue) staining of the patient.
D Lectin staining of a blood smear of a control (green).
E Lectin staining of a blood smear of the heterozygous mother (green).
F Lectin staining of a blood smear of the patient (green).
G Digital enlargement of an image area of the control shown in Fig EV2A to show presence of AAL positive platelets (green).
H Pappenheim staining of control to show presence of platelets.
I Digital enlargement of an image area of the heterozygous mother shown in Fig EV2B to show presence of AAL positive platelets (green).
J Pappenheim staining of control to show presence of platelets.
K Digital enlargement of an image area of the control shown in Fig EV2C to show lack of AAL positive platelets (green).
L Pappenheim staining of control to show presence of platelets.
A1 Merge of lectin (green) and DAPI (blue) staining of the heterozygous mother immediately before the first dose of fucose.
B1 Merge of lectin (green) and DAPI (blue) staining of the patient immediately before the first dose of fucose.
C1 Digital enlargement of an image area of the heterozygous mother shown in Fig EV2A1 to show presence of AAL positive platelets (green).
D1 Digital enlargement of an image area of the patient shown in Fig EV2B1 to show lack of AAL positive platelets (green).
E1 FACS analysis of patient (red) and control (green) PBMCs stained with 1 μg/ml FAA lectin without (dark) or with 200 mM L-fucose (light) before the start of fucose therapy.
A2 Merge of lectin (green) and DAPI (blue) staining of the heterozygous mother after 2 weeks of fucose therapy.
B2 Merge of lectin (green) and DAPI (blue) staining of the patient after 2 weeks of fucose therapy.
C2 Digital enlargement of an image area of the heterozygous mother shown in Fig EV2A2 to show presence of AAL positive platelets (green).
D2 Digital enlargement of an image area of the patient shown in Fig EV2B2 to show lack of AAL positive platelets (green).
E2 FACS analysis of patient (red) and control (green) PBMCs stained with 1 μg/ml FAA lectin without (dark) or with 200 mM L-fucose (light) 2 weeks after the start of fucose therapy.
A3 Merge of lectin (green) and DAPI (blue) staining of the heterozygous mother after 4 weeks of fucose therapy.
B3 Merge of lectin (green) and DAPI (blue) staining of the patient after 4 weeks of fucose therapy.
C3 Digital enlargement of an image area of the heterozygous mother shown in Fig EV2A3 to show presence of AAL positive platelets (green).
D3 Digital enlargement of an image area of the patient shown in Fig EV2B3 to show presence of AAL positive platelets (green).
E3 FACS analysis of patient (red) and control (green) PBMCs stained with 1 μg/ml FAA lectin without (dark) or with 200 mM L-fucose (light) 4 weeks after the start of fucose therapy.
A4 Merge of lectin (green) and DAPI (blue) staining of the heterozygous mother after 8 weeks of fucose therapy.
B4 Merge of lectin (green) and DA.PI (blue) staining of the patient after 8 weeks of fucose therapy.
C4 Digital enlargement of an image area of the heterozygous mother shown in Fig EV2A4 to show presence of AAL positive platelets (green).
D4 Digital enlargement of an image area of the patient shown in Fig EV2B4 to show lack of AAL positive platelets (green).
E4 FACS analysis of patient (red) and control (green) PBMCs stained with 1 μg/ml FAA lectin without (dark) or with 200 mM L-fucose (light) 8 weeks after the start of fucose therapy.

Data information: Green: *Aleuria aurantia* lectin; Blue: DAPI. PBMCs are highlighted by a white arrow in panel E. Platelets are highlighted by white or black arrows. Pappenheim images were taken with a 40x magnification. No lectin staining of platelets was visible in the blood smear of the individual (K). Pappenheim staining of the patient's blood showed presence of platelets (L). Scale bars = 50 μm with exception of C1, D1, C2, D2, C3, D3, C4, D4, here 10 μm scale bars are shown. Results of flow cytometry analysis of patient (red) and control (green) PBMCs stained with 1 μg/ml FAA lectin without (dark) or with 200 mM L-fucose (light) before the start of fucose therapy.

© 2021 The Authors

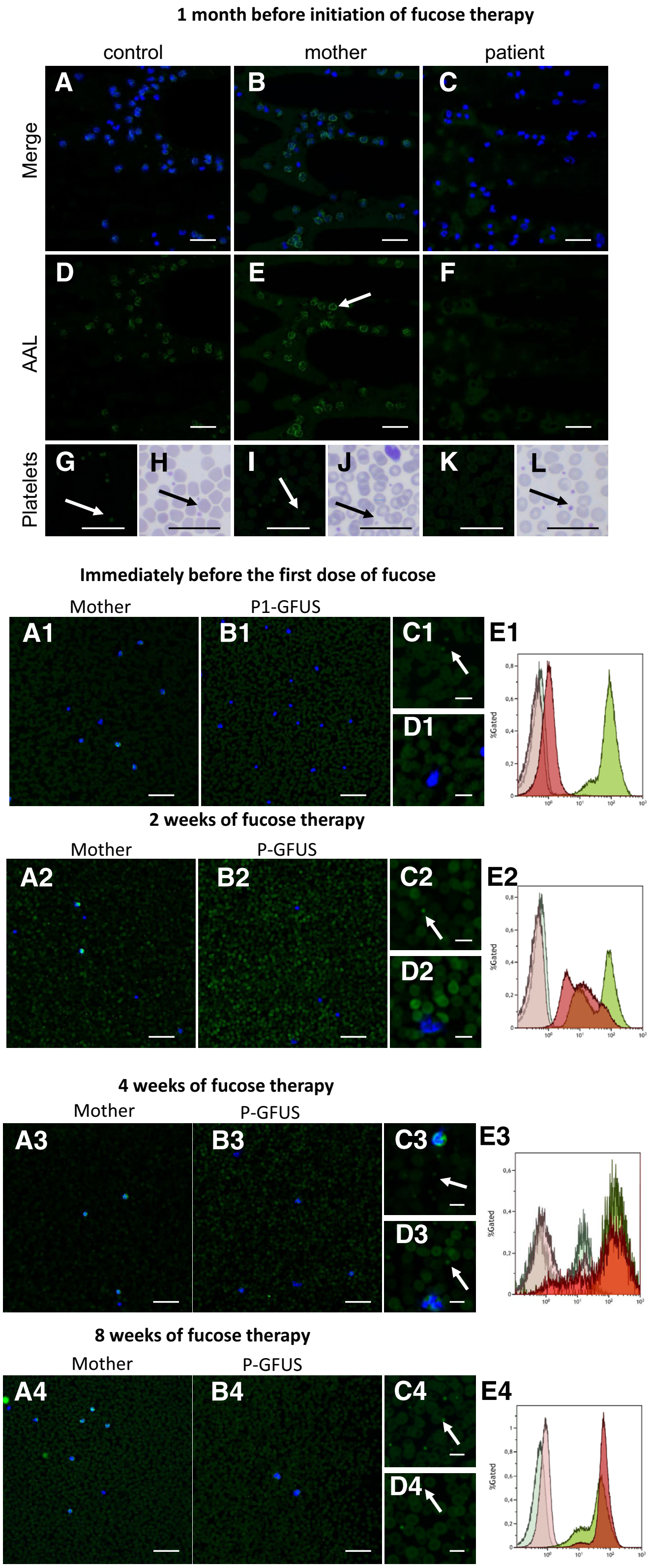


**Figure EV2.**

 © 2021 The Authors

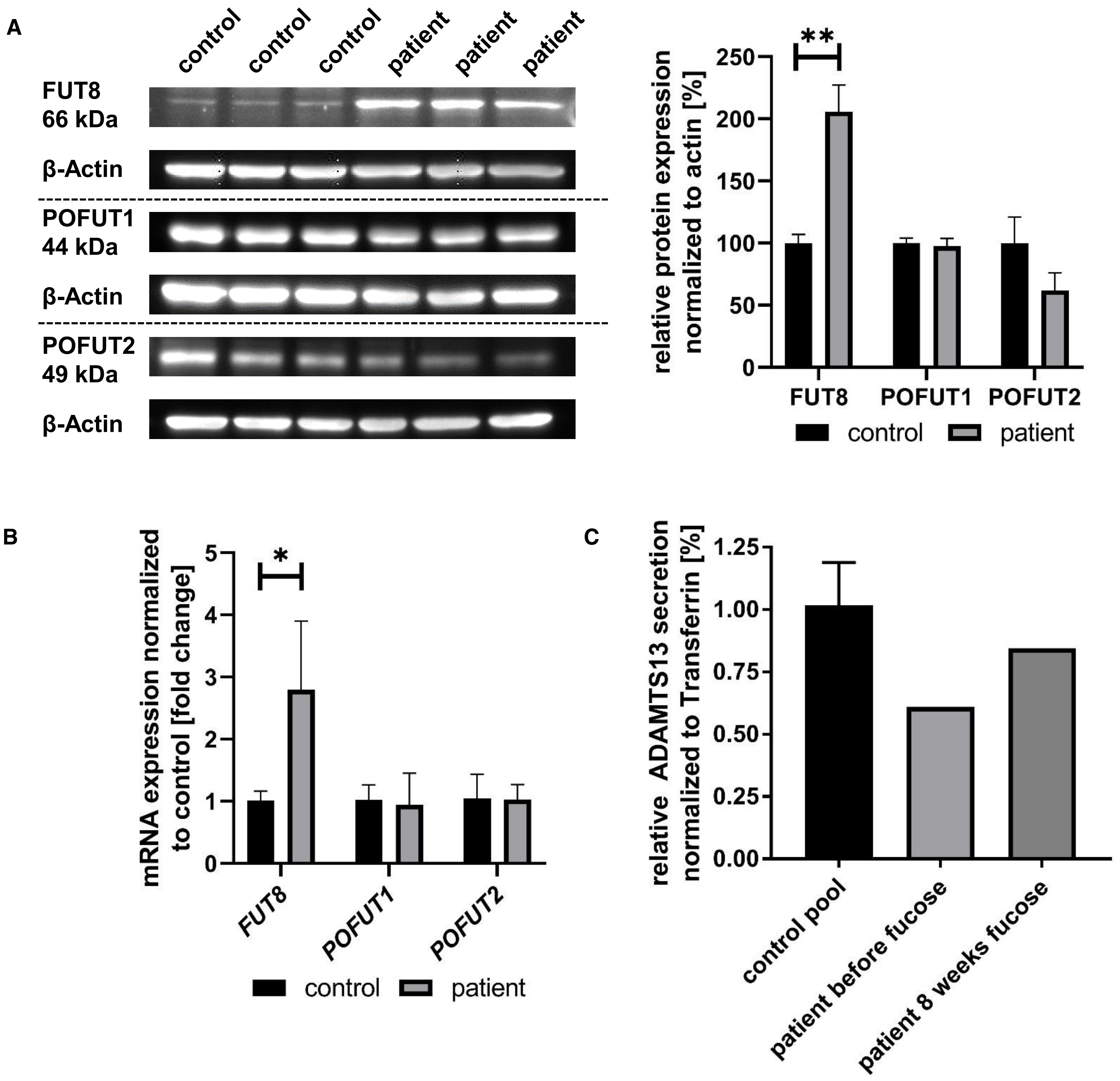


**Figure EV3. Effects of GFUS deficiency on the expression of fucosyltransferases and ADAMTS13.**

A Western blot analysis of FUT8, POFUT1 and POFUT2. Expression of the proteins was analysed by western blotting on a 10% SDS–PAGE with cytosolic fractions derived from control and patient fibroblasts. Data were obtained from fibroblasts; $n = 3$, for statistics an unpaired *t*-test was performed.

B qPCR studies on *FUT8*, *POFUT1* and *POFUT2*. mRNA expression revealed a significantly increased transcript level of *FUT8* (2.79 fold change; ± 1,10 fold change; $^{*}P = 0.0498$) normalized to a control. Expression of *POFUT1* and *POFUT2* was not changed significantly. Data were obtained from fibroblasts; $n = 9$; experiment was independently repeated three times, for statistics an ANOVA was performed.

C Expression of ADAMTS13 in sera. The amount of ADAMTS13 secreted into sera of controls and the patient before and after 8 weeks of L-fucose treatment was detected by western blot and normalized to transferrin. Data were obtained from serum; $n = 12$ for controls.

Data information: $^{*}P < 0.05$; $^{**}P < 0.01$. Bars and error bars represent mean ± SD. Exact *P*-values are reported in the results part.

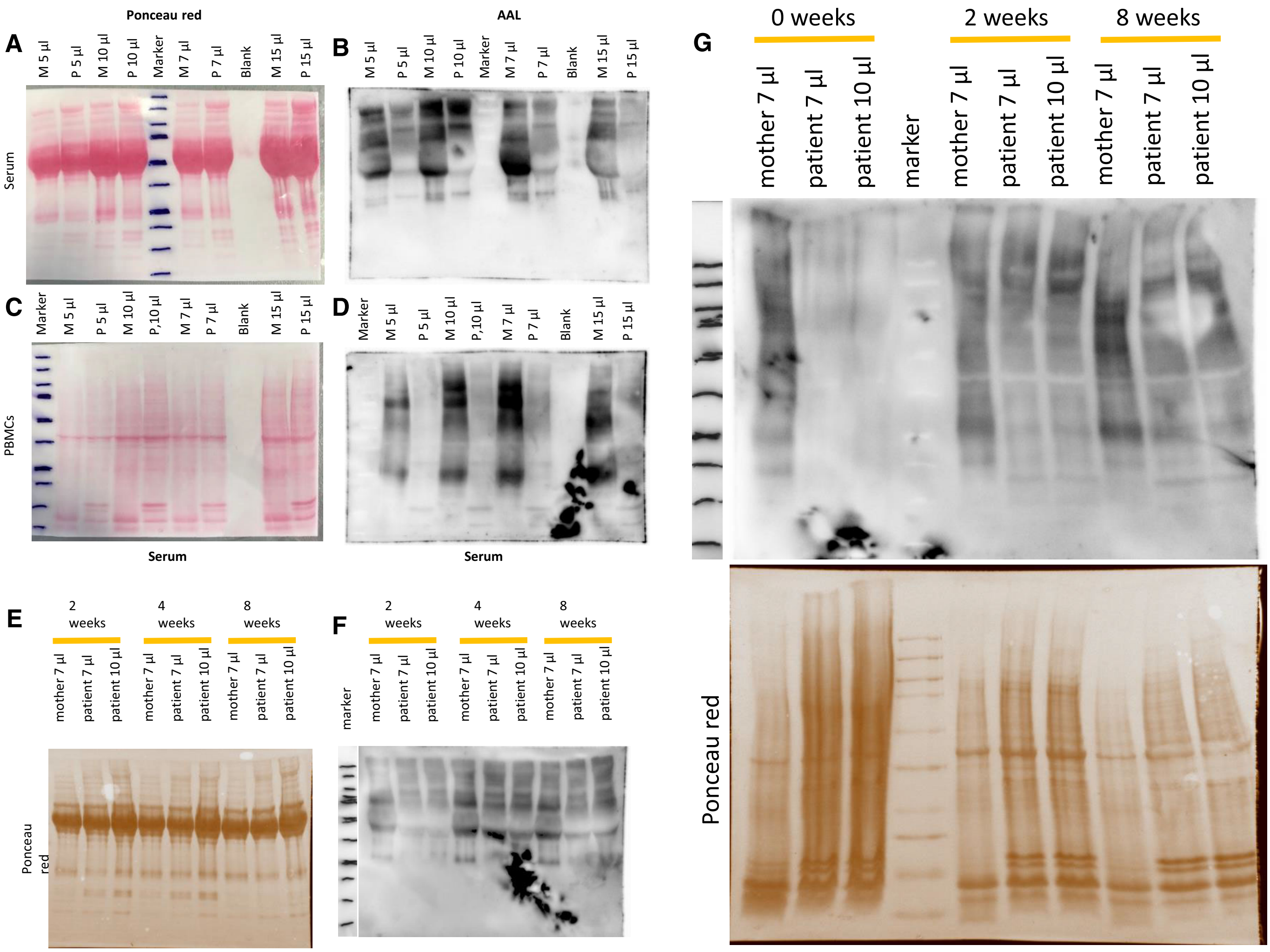


**Figure EV4.** ***Aleuria aurantia*** **lectin staining of proteins of serum and PBMCs from affected individual and her mother.**

A–D Blood samples were taken 2 months prior to therapy initiation. (A, C) Ponceau red staining which was used as loading control. In addition, different volumes were separated for better comparability. (B, D) Blots probed with the *Aleuria aurantia* lectin for visualization of core-fucosylated proteins.

E, F Lectin staining of serum proteins taken 2, 4 and 8 weeks after start of fucose therapy. (E) Ponceau red staining which was used as loading control. In addition, different volumes were separated for better comparability. (F) Blots probed with the *Aleuria aurantia* lectin for visualization of core-fucosylated proteins.

G Lectin blots revealed a severe reduction of protein fucosylation in patient serum and PBMCs.

Data information: A severe reduction of fucosylated proteins is present in serum and PBMCs of the affected individual compared to her mother 2 month before start of fucose treatment. M: mother; P: affected individual.
Source data are available online for this figure.

 © 2021 The Authors
