# Supplementary material for: A spoonful of L‐fucose—an efficient therapy for GFUS‐CDG, a new glycosylation disorder
Source: EMBO Mol Med. 2021 Sep 1;13(9):e14332. doi: 10.15252/emmm.202114332 (PMC8422078; doi:10.15252/emmm.202114332)
Supplement: Supplementary file 5 — Source Data for Expanded View [file EMMM-13-e14332-s005.zip › emmm202114332-sup-0005-SDataFigEV4 .pptx]

## Slide 1
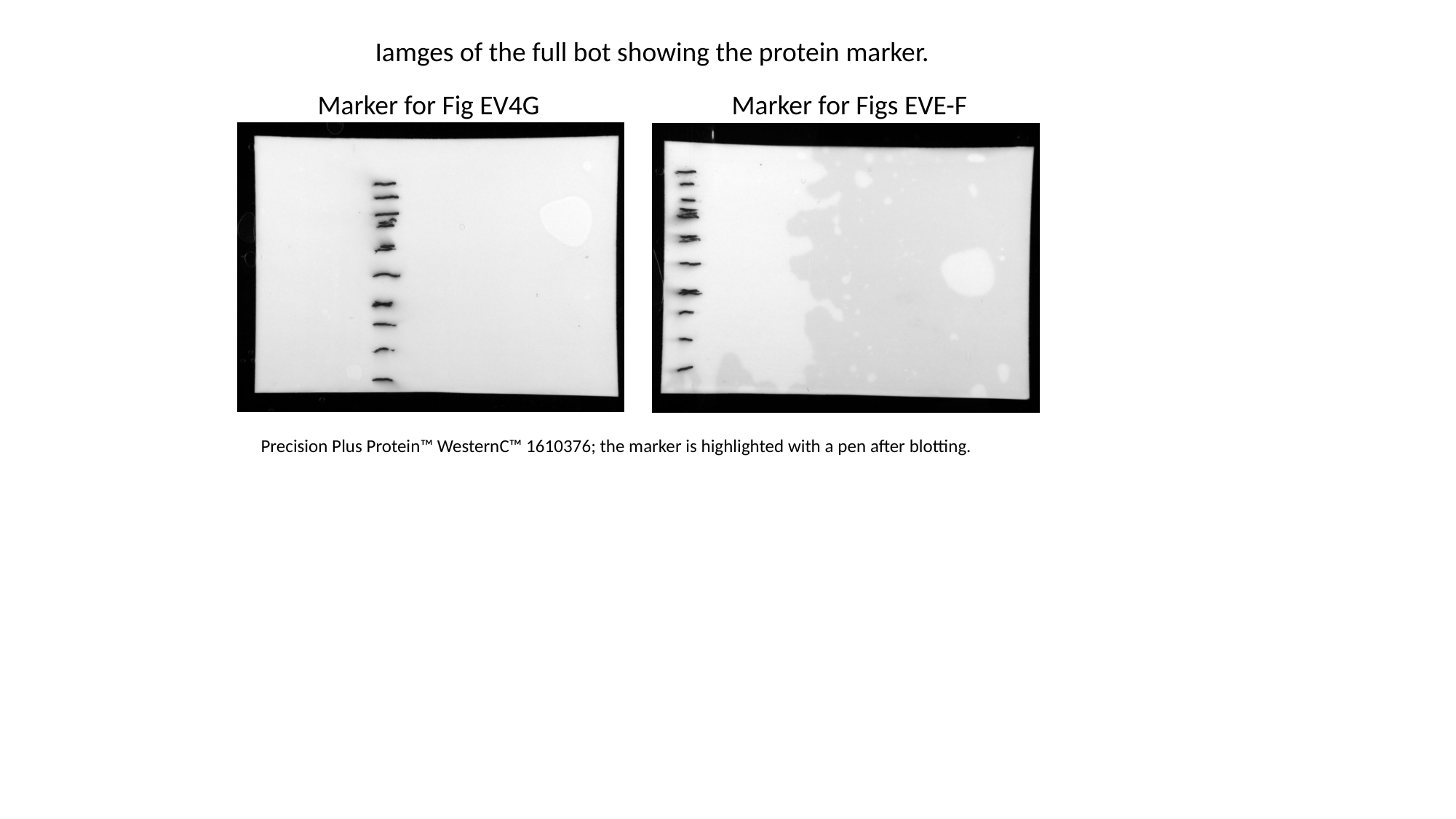

Iamges of the full bot showing the protein marker.
Marker for Fig EV4G
Marker for Figs EVE-F
Precision Plus Protein™ WesternC™ 1610376; the marker is highlighted with a pen after blotting.
